# A Rapid Test for Soy Aeroallergens Exposure Assessment

**DOI:** 10.1371/journal.pone.0088676

**Published:** 2014-02-12

**Authors:** Daniel Álvarez-Simon, María-Jesús Cruz, María-Dolores Untoria, Xavier Muñoz, Joan R. Villalbí, Ferran Morell, Susana Gómez-Ollés

**Affiliations:** 1 Servicio de Neumología, Hospital Universitario Vall d'Hebron, Barcelona, Spain; 2 Departament de Medicina, Universitat Autònoma de Barcelona, Barcelona, Spain; 3 CIBER Enfermedades Respiratorias (CIBERES), Barcelona, Spain; 4 Departament de Biologia Cel·lular, de Fisiologia i d'Immunologia, Universitat Autònoma de Barcelona, Barcelona, Spain; 5 Agència de Salut Pública de Barcelona, Barcelona, Spain; 6 Centro de Investigación Biomédica en Red de Epidemiología y Salud Pública (CIBERESP) & Institut d'Investigació Biomèdica Sant Pau, Barcelona, Spain; New York University, United States of America

## Abstract

**Background:**

Determining soy aeroallergens levels is extremely important in the assessment of health risks due to these airborne substances. Currently, soy aeroallergens exposure in the environment is monitored using enzyme immunoassays (EIA) which must be evaluated in a specialized laboratory by skilled personnel.

**Objective:**

To describe the development and performance of a rapid immunochromatography assay for the detection of soy aeroallergens in environmental samples.

**Methods:**

A test strip using gold labeled anti-soy hull low molecular weight extract (SHLMWE) antibody for the rapid detection of soy aeroallergens in environmental samples was developed. One hundred nineteen airborne samples were analysed in parallel by the strip assay and the anti-SHLMWE sandwich EIA. The assay results were visually analysed by three independent observers who ranked samples as: -, + or ++. Strips were also scanned and analysed by densitometry.

**Results:**

The rapid test detected a range of concentrations from 6.25 to 25 ng/mL. Agreement in strip assay interpretations between evaluators was substantial (Kappa = 0.63; CI 0.544–0.715). Visual interpretation also gave a good concordance with EIA results, with sensitivity ranging from 77.3 to 100 and specificity from 65 to 83.5 depending on the observer. Furthermore, a strong correlation was observed between densitometry results of strip assay and EIA determinations.

**Conclusions:**

The strip assay developed is rapid, simple, and sensitive and does not require expensive equipment or specific skills. It has considerable potential in the environmental monitoring field for screening soy aeroallergens levels in port cities where allergen measurements are not currently performed. Due to its simplicity, the test will improve the management of soy allergic patients by controlling environmental allergen exposure without the need for apparatus or skilled personnel.

## Introduction

Soy dust is a well-known aeroallergen. In cities with ports where soybeans are loaded or unloaded, community outbreaks of asthma have been recorded and attributed to inhalation of soy dust [1]–[5]. In addition, exposure to soy dust in the workplace has been identified as a cause of occupational asthma [6]–[8] and hypersensitivity pneumonitis [9].

Avoiding or reducing exposure to inhaled soy allergens is crucial in order to prevent the adverse respiratory outcomes associated with soy exposure. Measuring soy aeroallergens levels is vital in order to assess the health risks and the efficacy of current exposure reduction measures, and to establish whether further additional measures are needed. A striking example is the city of Barcelona, Spain, where considerable efforts have been made to improve the control of soybean dust released during harbor activities. Control measures adopted include the assessment of the emission and dispersion of the allergen, the identification of allergen concentration levels compatible with health, the reduction of allergen emission levels, and the definition of complementary safety measures. Thanks to this strategy, this important economic activity is compatible with the strict requirements the city maintains for public health [10].

One of the measures adopted in Barcelona is daily soy monitoring in a district close to the harbor using a large-volume automated air sampler, as a means of determining the exposure in the population [11]. From autumn 1997 to May 2012 these measurements were performed by an enzyme immunoassay (EIA) inhibition assay using a serum pool with specific immunoglobulin E (IgE) antibodies from subjects allergic to soybean as a detector antibody [10], [11]. Levels of soy aeroallergens should not exceed two threshold values: the Environmental High Threshold Limit Value (EH-TLV, set at 480 U/m^3^), and the Environmental Low Threshold Limit Value (EL-TLV, set at 160 U/m^3^). These threshold values were determined empirically based on the levels reached during epidemic and non-epidemic days [12]. When the EL-TLV is exceeded, soy facilities and meteorological conditions are assessed and a report is produced, and when the EH-TLV is reached an inspection of the soy facilities' processes is performed [12]. Since April 2012, monitoring of soy aeroallergens exposure in the city has been performed by a sandwich EIA described previously [13] and the EH-TLV and EL-TLV were redefined as 19 ng/m^3^ and 6 ng/m^3^ respectively. This method of environmental monitoring requires the use of a specialized laboratory staffed by skilled personnel.

The Barcelona experience shows that industrial soybean plants may be safely located near urban settings if strict control criteria are applied, including assessment of soy aeroallergens levels. The identification of similar problems in port cities with soybean harbor facilities in Spain [2], [4], [5], and in other countries [3] highlights the importance of the monitoring process. However, to date, no country has developed legislation to regulate this environmental risk [10], partly due to the lack of a widely available assay to monitor soy aeroallergens levels. Clearly, cost-efficient and less labor-intensive technological procedures for monitoring soy allergens levels are needed.

The aim of the present study is to describe the development and performance of a rapid immunochromatography assay for the detection of soy aeroallergens in environmental samples. The result of the test appears as a distinct color band in the “test line” of the soy strip. The test is simple and straightforward, the results are easily interpretable, and neither expensive equipment nor specific skills are required.

## Materials and Methods

### Preparation of immunoassay reagents

#### a) Preparation of Soy Hull Low Molecular Weight Extract (SHLMWE)

As previously described [13] the SHLMWE was obtained from soy hull with a chromatographic process (CM-cellulose and DEAE-cellulose chromatography). The SHLMWE contains low molecular weight allergens responsible for the asthma outbreaks in Barcelona [13]. The protein concentration in the extract was 1 mg/mL, as determined by the bicinchoninic acid (BCA) method (Pierce Chemical Co., Rockford, IL, USA) following the manufacturer's instructions.

#### b) Production of anti-SHLMWE polyclonal antibodies and colloidal gold labeling

Anti-SHLMWE polyclonal antibodies were produced as previously described [13]. The IgG fraction of the polyclonal antiserum was isolated on an immobilized Protein A–agarose column (Pierce), and then eluted onto an Excellulose column (Pierce) to desalt and exchange the buffer to Phosphate buffered saline (PBS). The protein concentration of the IgG fraction was 3.3 mg/mL, as determined by the Bicinchoninic acid (BCA) method (Pierce). No cross-reaction was observed with other legumes or cereals tested (data not shown).

A portion of Anti-SHLMWE polyclonal antibody was colloidal gold labeled by BBI Solutions (British Biocell International, Cardiff, UK). Briefly, the antibody was conjugated to a 40 nm gold colloid using passive adsorption and was stable at least for 10 months. After incubation with rabbit Anti-SHLMWE polyclonal antibodies, the conjugate was blocked with bovine serum albumin. The resulting conjugate was concentrated by ultrafiltration to give a final optical density (OD) (520 nm) of 10 and suspended in a 2 mM disodium tetraborate buffer pH 7.2, containing 0.095% sodium azide.

### Rapid immunochromatographic test strip

#### a) Membrane blotting and assembly of the immunostrip

**Figure 1:**
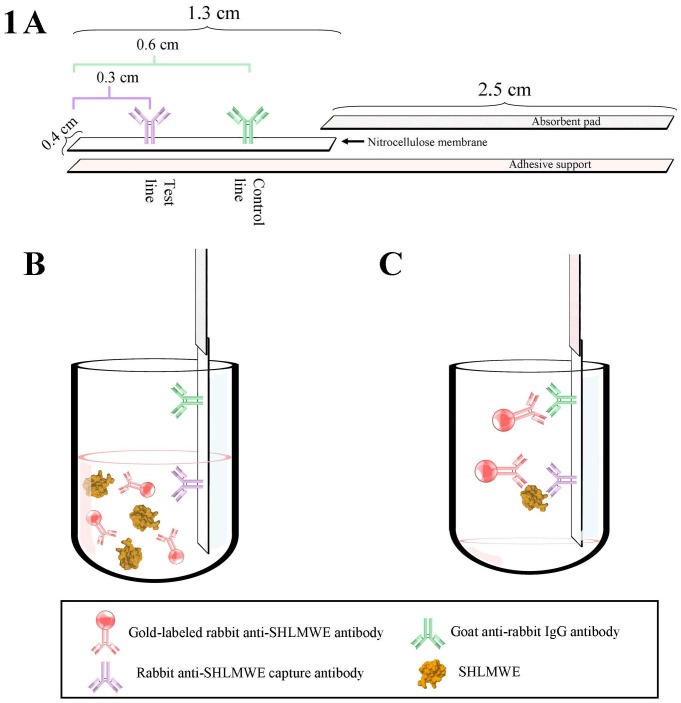
Schematic diagram of the strip assay: 1A) Assembly of the immunostrip; 1B) Dipping of the strip; 1C) Binding of gold labeled antibody and gold labeled antibody-SHLMWE complex to control and test line respectively.

The rabbit anti-SHLMWE (1200 ng/strip) capture antibody was applied to direct cast backed nitrocellulose nitrate membranes (Unisart CN95 19910 Sartorius Stedim Biotech, Goettingen, Germany) in a line format at 3 mm from the application end of the strip (Test line). At 6 mm, a goat anti-rabbit IgG antibody (Southern Biotechnology Associates, Inc., Birmingham, AL, USA) was bound at 600 ng/strip to provide a positive control (Control line). Membranes were dried for 1 h at 37°C, and assembled as follows: on an adhesive support an absorbent pad was layered and the membrane was placed overlapping it by 2 mm and cut into individual strips of 4 mm in width (see schematic diagram in figure 1A).

#### b) Rapid immunochromatographic test procedure

After optimization, environmental samples were diluted 1:100 in sample diluent buffer (PBS,1% BSA,0.5% Tween 20). Twenty microliters of diluted samples or SHLMWE standards were mixed 1:1 in a microtiter plate well with a gold labeled anti-SHLMWE antibody solution with an OD of 2. During brief gentle mixing, the gold-labeled antibody was allowed to react with soy allergens in the sample to form a complex. The test strip was dipped in the well and allergen-antibody complexes diffused across the nitrocellulose membrane and reacted with the specific anti-SHLMWE antibodies immobilized on the test line forming a red-purple line, or migrated further and reacted with the goat anti-rabbit IgG antibody in the control line. Figures 1B and 1C provide a schematic diagram of the rapid immunochromatographic test procedure.

**Figure 2:**
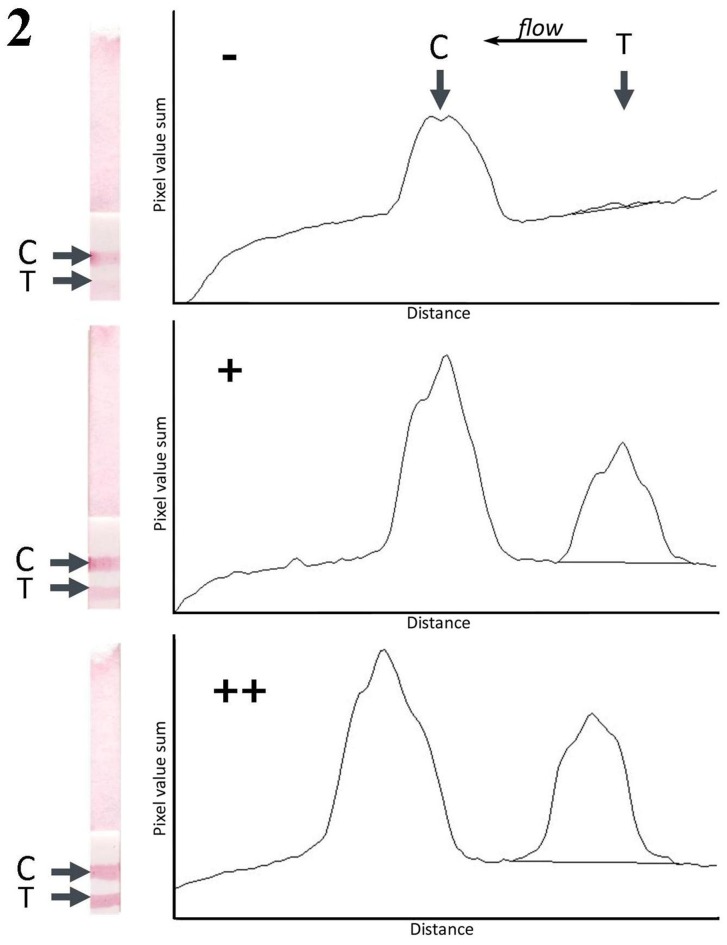
Three-point standard curve of SHLWE used as a source for comparison to rank the line seen on the strips as negative, positive or double positive. The figure shows scanned strips and density analysis of the strips.

Strip assay results were read by three independent observers 30 minutes after application of sample. A visible red-purple band had to appear at the control line for the test to be valid. After the liquid reagent flowed through the lines, the changes in the color on the test lines could be observed with the naked eye. Readers ranked sample results as negative, positive or double positive by comparing the color intensity of the test line with the test line of a 3-point standard curve: 1.56 ng/ml (−), 6.25 ng/ml (+) and 25 ng/ml (++) (Fig. 2). In parallel, test lines were scanned with a flatbed scanner (Epson Expression 1600) and the line intensities were analysed by densitometry with ImageJ 1.45s (NIH, USA).

**Figure 3:**
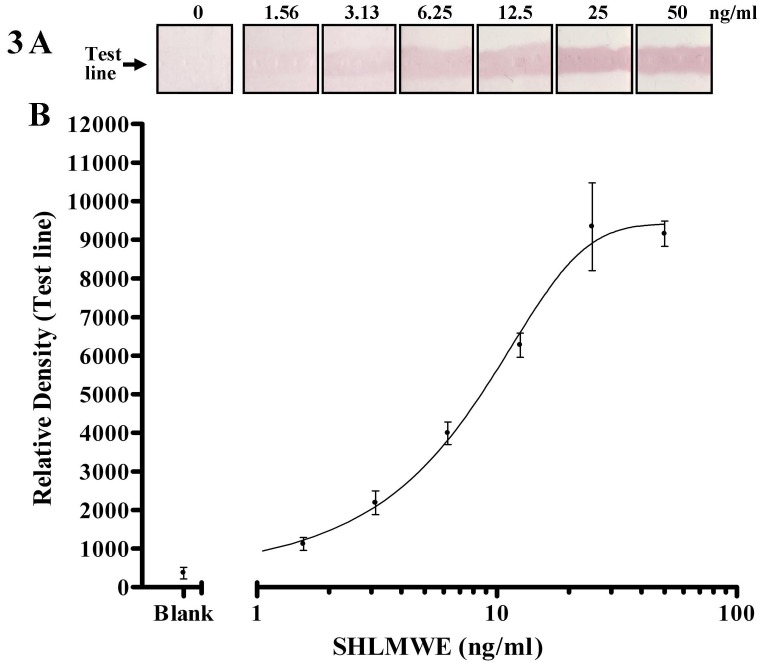
A typical strip assay standard curve to determine the limit of detection of the assay: 3A) Scanned strips at test line level; 3B) Graph of densitometry results (means ± SEM) at the indicated concentrations of SHLMWE using a four parameter logistic curve fit.

To determine the limit of detection of the strip assay, SHLMWE was used in a series of concentrations from 1.56 to 50 ng/mL and tested in triplicate (Fig. 3).

### Anti-SHLMWE sandwich EIA

Soy aeroallergens levels were measured by the anti-SHLMWE sandwich EIA described previously [13]. Results directly obtained by the EIA are expressed in nanograms per milliliters, referring to the protein content of the standard preparation. However, results of soy environmental levels are expressed as nanograms per cubic meter (ng/m^3^) of air. As previously mentioned, for surveillance of soy aeroallergens exposure in the city of Barcelona using the sandwich EIA, an EH-TLV of 19 ng/m^3^ and a EL-TLV of 6 ng/m^3^ were defined, corresponding to 627 ng/ml and 198 ng/ml respectively for an average air volume of 165 m^3^.

### Sample collection and analysis

One hundred nineteen samples routinely used for the daily monitoring of soy aeroallergens in the city were studied. Briefly, samples were collected from the air of the city of Barcelona with a large-volume automated air sampler (CAV-A/HF, MCV, SA, Barcelona, Spain) containing glass microfiber filters with a 1μm pore size (Whatman International Ltd., UK), installed near the harbor and changed daily as previously described [12], [14]. Dust was collected at a flow of 55 m^3^/h. Soy aeroallergens were extracted from a eighth of the filters in 5 mL PBS/0.2% BSA/0.1% Tween 20 (pH 7.4) overnight at 4°C. Filters were discarded and the eluates stored at −20°C. All the eluates were analysed in parallel by the anti-SHLMWE sandwich EIA and the rapid immunochromatographic strip test.

### Statistical analysis

The percentage of samples higher than the settled EH-TLV and the median and range of soy aeroallergens levels analysed by the EIA were calculated. A one-sample Kolmogorov-Smirnov test to assess normality was calculated for soy aeroallergens levels measured by EIA. The Kolmogorov-Smirnov test showed a non-normal distribution; therefore, the correlation between airborne soy allergen levels and strip assay density values were analysed using Spearman's rank correlation coefficient (r_s_). Differences between density values of samples categorized by the EIA assay as higher or lower than the EH-TLV were analysed by the Mann-Whitney test. Differences were considered significant at a p-value of ≤0.05.

The assay results were visually analysed by three independent observers. Agreement between the three evaluators was calculated using the Fleiss' Kappa test and percentage of agreed interpretations. Sensitivity, specificity and percentage of agreement of visual interpretation of the strips versus EIA results was calculated for each reader and differences between soy allergen levels of samples visually categorized as negative, positive or double positive were analysed by the Kruskal-Wallis test followed by Dunn's multiple comparison test.

Statistical analyses were performed using GraphPad Prism version 4.01 for Windows, (GraphPad Software, San Diego, California, USA) and a freely-available Microsoft Excel spreadsheet that estimates the generalized kappa statistic based on equations presented in Fleiss et al. [15].

## Results

### Lower and upper limit of detection

The rapid test detected a range of concentrations from 6.25 to 25 ng/mL (Fig. 3). The lower limit of detection (LOD) of the strip assay was determined as the minimum amount of SHLMWE producing a clearly visible red-purple band at the test line (6.25 ng/ml, Fig. 3). The upper LOD of 25 ng/ml was determined as the maximum amount of SHLMWE producing a more intense red-purple band than the preceding concentration at the test line (Fig. 3).

### Sample dilution

In the EIA, the median (range) (ng/ml) was 274.5 (1.18–1848), 22 (18.5%) samples being higher than the settled EH-TLV of 627 ng/ml. In the strip assay, samples were diluted 1/100 to match the lower LOD of the strip assay of 6.25 ng/ml with the environmental settled high threshold value. Thus, a positive or double positive result in the strip assay would indicate that the sample has a soy aeroallergens concentration equal to or higher than the settled EH-TLV.

### Inter-reader agreement and comparison between the EIA and the strip assay

**Figure 4:**
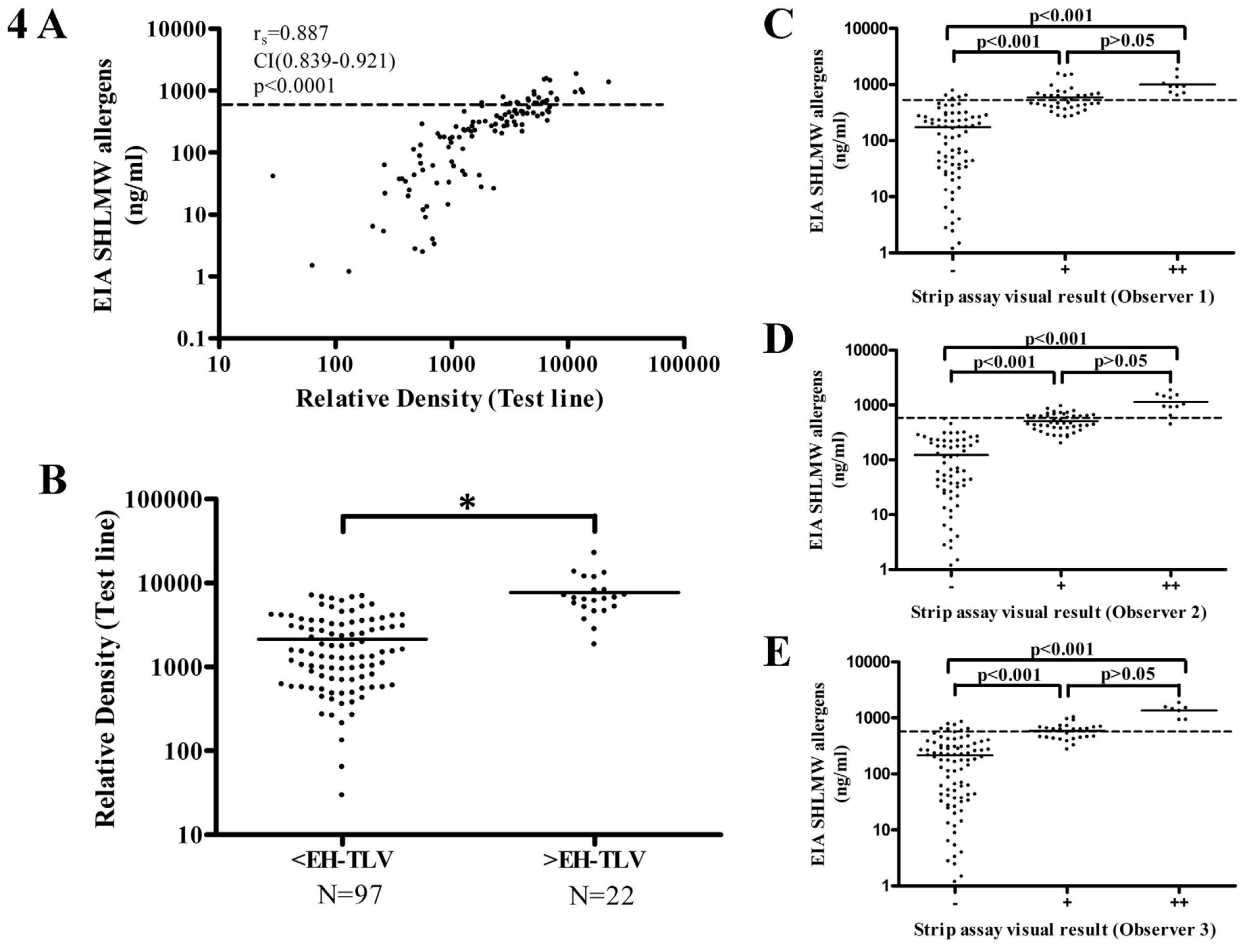
Densitometry results of strip assay and EIA determinations and EIA results by strip assay visual interpretation category. The dashed lines indicate the EH-TLV of 627 ng/ml. 4A) Scatter plot showing the correlation between SHLMW allergens concentrations and density values of strip assay; 4B) Densitometry results by EIA result category (higher or lower than the EH-TLV).* p<0.0001; EIA results by strip assay visual interpretation category: 4C) Observer 1; 4D) Observer 2; 4E) Observer 3.

The three observers agreed on the interpretation of 71.4% of the samples, and in all the samples at least two readers agreed. Ranking samples as negative, positive or double positive, agreement in strip assay interpretations between evaluators was substantial, with a kappa index of 0.63 (CI 0.544–0.715). The kappa index was higher (0.701, CI 0.598–0.805) when positive and double positive results were considered as one category. In addition, strips were scanned and the color intensity of the test line was analysed by densitometry. The values calculated by ImageJ are essentially arbitrary and only have meaning within the context of the set of peaks that are analysed together. Thus, all samples were analysed at the same time. A strong correlation was observed between the densitometry results of strip assay and EIA determinations (r_s_ =  0.887 [CI 0.839–0.921]; p<0.0001) (Fig 4A). Besides, intensity of the line analysed by densitometry was significantly higher (p<0.0001) in the samples categorized by the EIA assay as higher than the EH-TLV (Fig 4B). Visual interpretation of the strip assay also had a good concordance with EIA results, with sensitivity ranging from 77.3 to 100 and specificity from 65 to 83.5 depending on the observer (Table 1). The percentages of agreement were 75.63%, 71.43% and 82.35% for observers 1, 2 and 3 respectively. Samples visually categorized as negative by strip assay had significantly lower soy allergen levels by sandwich EIA and samples categorized as double positive had significantly higher levels than those categorized as negative or positive by all readers (Figures 4C, 4D and 4E). The three readers visually detected SHLMW allergens in a concentration range from 266 to 1848 ng/mL, 200.4 to 1848 ng/mL and 274.5 to 1848 ng/mL.

**Table 1:** Sensitivity and specificity of strip assay estimated from the visual interpretation of three independent observers.

	Sensitivity; %(CI)	Specificity; %(CI)
**Observer 1**	86.4 (65.1–97.1)	73.2 (63.2–81.7)
**Observer 2**	100 (84.6–100)	65 (54.6–74.4)
**Observer 3**	77.3 (54.6–92.2)	83.5 (74.6–90.3)

## Discussion

Surveillance of soy aeroallergens levels in the harbor areas of cities where soybean is loaded or unloaded and/or processed is crucial in order to prevent asthma outbreaks [10], [11], [16]. Thus, there is a clear need for broadly available, fast, easy-to-use devices for detection of high levels of soy aeroallergens in the air of port cities. To address this need, we developed a strip assay able to measure soy aeroallergens at levels as low as 6.25 ng/ml.

The LOD of the strip assay is quite similar to those described for other immunochromatographic assays developed for allergens from house dust mites with a sensitivity of 1–2 ng/ml [17], for fungal alpha-amylase, in which the LOD was 1–10 ng/ml [18] or for rodents, in which the LOD was 31 pg/ml for both mouse and rat urinary allergens, though a LOD of 4 ng/ml for rat urinary allergens has been indicated as more realistic for real field samples [19].

Surprisingly, the readers detected soy aeroallergens concentrations below the LOD of the assay determined using a buffer system. The actual LOD of the assay with field samples ranged from 2 to 2.7 ng/ml depending on the reader. This observation has also been reported in other studies. For example, in some samples Tsay et al. [17] graded line intensity as medium or high although mite group 2 levels analysed by ELISA were below the theoretical sensitivity of the rapid test. The same occurred in the Bogdanovic et al. [18] study, where some samples with α-amylase levels below the sensitivity of the rapid test were classified as positive.

In a buffer system with a concentration of SHLMW allergens above 1,600 ng/mL the assay signal, i.e., the gold colloid line intensity, decreased as a result of the prozone or high-dose hook effect (results not shown). This effect appears in one-step immunoassays where sample and labeled antibody are added simultaneously and the antibodies are saturated by a very high concentration of sample antigen binding to all available sites and preventing the sandwich-formation. Thus, the hook effect causes false-negative results [20]. To detect the hook effect, samples are often tested undiluted and after dilution. Unfortunately, this approach increases labor and reagent costs for assays that only rarely encounter extremely high antigen concentrations. Fortunately this effect will not have any significance for the control of environmental samples near the harbor of Barcelona as in recent years the highest concentration reached was 1,848 ng/ml. This is also the case of the samples analysed for this study; the highest SHLMW allergen concentration was 1,848 ng/ml, and it was tested at 1:100 dilution. Thus, in this study we did not observe the hook effect. However, for each new sampling site without previous recordings of soy aeroallergens levels, we recommend performing an initial evaluation with the sandwich EIA assay and the subsequent follow up evaluations with the strip assay at an adequate dilution based on the EIA results.

Visual interpretation of the strip assay is inherently subjective as it depends, among other factors, on the reader's perception of color. Thus, it is important to assess inter-rater agreement. In this study agreement between readers was substantial according to the classification recommended by Landis and Koch [21]. Despite the limitations of visual interpretation, the results of the rapid test presented high agreement with the EIA results, and strip assay visual interpretation showed a high sensitivity and specificity. Furthermore, line intensity, analysed by densitometry (an objective measurement) of samples categorized as negative was significantly lower than the line density of positive and double positive samples (data not shown).

Environmental monitoring to assess airborne allergens is a time-consuming process that commonly includes three steps: sampling aeroallergens on a filter, elution of the allergens, and determination of allergen levels by an allergen-specific EIA. The strip assay replaces the EIA, speeding up the last step of the process. The strip assay's advantage, its speed, is not decisive at present for airborne samples as the time needed to obtain the sample is still too long. In fact, this speed will be more useful for industrial hygiene monitoring than for environmental monitoring, as some of the sampling procedures widely used in occupational hygiene take only a short time (for example, surface wipe sampling, dust sampling or bulk sampling).

In conclusion, the strip assay described is rapid, simple, sensitive and does not require expensive equipment or specific skills. Thanks to its simplicity, this method has considerable potential in the field of environmental monitoring for screening soy aeroallergens levels in port cities with soybean harbor facilities where allergen levels are not currently measured.
